# A Distributed Oracle Using Intel SGX for Blockchain-Based IoT Applications

**DOI:** 10.3390/s20092725

**Published:** 2020-05-10

**Authors:** Sangyeon Woo, Jeho Song, Sungyong Park

**Affiliations:** Department of Computer Science and Engineering, Sogang University, 35 Baekbeom-ro, Mapo-gu, Seoul 04107, Korea; tkddus121@sogang.ac.kr (S.W.); oidwin@sogang.ac.kr (J.S.)

**Keywords:** blockchain, blockchain oracle, ethereum, Internet of Things, smart contracts

## Abstract

A *blockchain oracle problem* is a problem that defines a mechanism for how to safely bring external data to the blockchain. Although there have been various research efforts to solve this problem, existing solutions are limited in that they do not support either data availability or data integrity. Furthermore, no solution has been proposed to minimize the response time when an oracle server is malicious or overloaded. This paper proposes a distributed oracle using Intel Software Guard Extensions (SGX). The proposed approach uses multiple oracle servers to support data availability. It also supports data integrity using Intel SGX and Transport Layer Security (TLS) communication. The reputation system, which favors oracle servers with short response times, minimizes the average response time even if some of the oracle servers are malicious. The benchmarking results show that the response time of the proposed approach with 3 oracle servers is only 14% slower than a centralized oracle called Town-crier and scales well even if the number of oracle servers is increased up to 9. The reputation system is also evaluated, and its feasibility is analyzed using various experiments.

## 1. Introduction

Blockchain is a peer-to-peer distributed ledger system where network participants own a ledger and validate transactions through a consensus algorithm [1]. Although the blockchain was originally developed as part of Bitcoin [1], it has recently been emerged as an innovative technology that can support a variety of fields such as healthcare [2], Internet of Things (IoT) [3] or medical data management [4]. With the introduction of smart contract in the blockchain, the application fields of the blockchain have become more diverse. A smart contract is a collection of code and data that is stored on a block, and its execution ensures consistency and integrity through consensus among participants. Using smart contract, many blockchain-based IoT decentralized applications (Dapps) such as Shipchain [5], Supplychain [6], Autoblock [7] have also been developed. Those Dapps usually require external IoT data to be brought into the blockchain.

One of the most important challenges in the blockchain-based IoT Dapps is how to bring external data into the blockchain, while guaranteeing the same level of security as the blockchain. This is called the *blockchain oracle problem* [8]. Blockchain is a Turing-complete machine which has deterministic output for internal data and input, but external data in the real world such as weather temperature or stock price is non-deterministic. When external data is brought into the deterministic blockchain, non-deterministic results can be created.

Furthermore, to develop secure and robust blockchain-based IoT Dapps, the *blockchain oracle* or *oracle*, a middle-ware that allows external data to be imported into the blockchain, should support *data availability* and *data integrity*. Data availability means that the requested external data must always be accessible. In other words, an oracle must ensure that it can provide data against internal errors or external malicious attacks. The blockchain oracle must also ensure data integrity. When an oracle provides external data to the blockchain, the integrity of the data should be guaranteed so that Dapp can execute flawlessly. In addition, in order to provide time-variant IoT data, an oracle must minimize *response time*, which is an interval between the time when a blockchain requests data and the time it receives data. Therefore, an oracle needs a method to minimize response time in order to prevent it from heavily fluctuating due to the limitation of node performance or a malicious node. Malicious oracle is a node that tampers data in the oracle or abuses data for its own benefit when importing external data to the blockchain.

Various oracle protocols have been proposed to address these challenges such as Oraclize [9], Town-crier [10], ASTRAEA [11]. Shintaku [12], and Chainlink [13]. However, existing oracle protocols are limited to develop secure and robust blockchain-based IoT Dapps in the sense that they are either centralized [14,15] or insecure when fetching external data [16,17,18]. Moreover, providing time-variant data that varies in value over time such as IoT data is almost impossible [19,20].

This paper proposes a distributed oracle, DiOr-SGX, which can safely import time-variant external data into the blockchain using Intel Software Guard Extensions (SGX) [21]. DiOr-SGX solves the problem of single point failure due to the centralization of a single oracle server by configuring multiple distributed oracle servers. Each oracle server in DiOr-SGX verifies data pulling procedure in which other oracle nodes pull data from external data sources through remote attestation provided by Intel SGX. In addition, DiOr-SGX solves data abusing problem that a malicious leader oracle server selectively sends external data to the blockchain for its own benefit through a reputation system. This paper makes the following specific contributions.

- Support for data availability: DiOr-SGX consists of multiple distributed oracle servers, where a leader oracle server is elected for collaboration among them. This ensures data availability even when a single oracle server fails.
- Support for secure oracle protocol using Intel SGX and TLS: DiOr-SGX guarantees data integrity through Intel SGX and Transport Layer Security (TLS) communication with external data sources. Since DiOr-SGX performs TLS communication for pulling external data inside the SGX enclave of each oracle server, data manipulation is impossible.
- Support for time-variant IoT data using reputation system: DiOr-SGX can provide time-variant IoT data into the blockchain. For time-variant IoT data, response time is important. DiOr-SGX elects a leader oracle server through a reputation system based on previous response times. This allows DiOr-SGX to provide a relatively consistent response time even if a malicious leader oracle server exists among multiple oracle servers.
- Real implementation: DiOr-SGX has been implemented over Ethereum blockchain [22] and its performance is compared with other competitors. The benchmarking results show that DiOr-SGX ensures response time even in an environment where a malicious oracle server exists, while the decrease in response time of 14% compared to the existing centralized oracles is minimal.

This paper is organized as follows. Section 2 describes background and research motivation of DiOr-SGX. Section 3 introduces various research efforts related to DiOr-SGX. Section 4 discusses the overall architecture of DiOr-SGX and its implementation issues in detail. Section 5 evaluates the performance of DiOr-SGX through experiments and justifies whether DiOr-SGX can address the problems presented in Section 2. Section 6 finally concludes this paper.

## 2. Background and Motivation

In this section, we briefly describe the background of Ethereum and smart contract, oracle problem, and Intel SGX. The motivation of DiOr-SGX is also discussed.

### 2.1. Ethereum and Smart Contract

Ethereum [22] is a permissionless blockchain platform for creating and executing Dapps through smart contract. A smart contract is an application code and data executed on all participating blockchain nodes, which ensures integrity and reliability of its execution results.

Ethereum provides users with the Turing-complete programming languages such as Solidity [23] or Serpent [24]. Ethereum users create a smart contract using those languages. The smart contracts created by users are compiled into the bytecode to be deployed in the blockchain network. As a deployed smart contract is considered to be an account, the contract can be executed in the similar way that users send a transaction to the account. The Ethereum Virtual Machine (EVM) is a 256-bit virtual machine (VM) that can execute the deployed smart contract. All nodes can execute the deployed smart contract by using the EVM. Therefore, based on the information in the smart contracts that are deployed through blockchain, all nodes can execute all smart contracts and validate the results executed by other nodes.

### 2.2. Blockchain Oracle Problem

**Figure 1:**
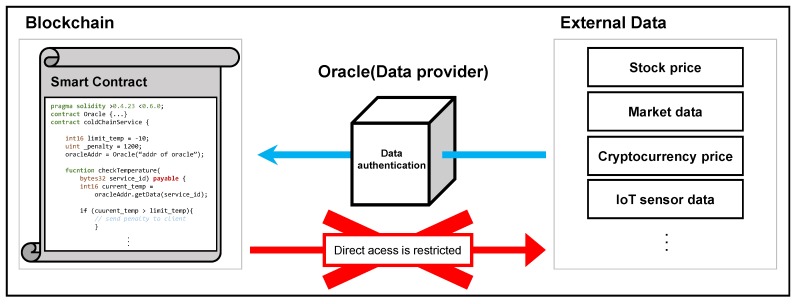
Blockchain Oracle problem.

The oracle problem [8] is an issue of how to bring real life external information such as stock price or market data to the blockchain so that smart contracts can execute based on them. Since the blockchain cannot directly access external data, a trusted third-party data provider called *oracle* is needed to transfer the information on behalf of the blockchain as shown in Figure 1. However, to bring such external data into the blockchain, the same level of security as the blockchain must be guaranteed when pulling external data. For example, assume that SportX is a sports betting Dapp running on Ethereum. In this case, this Dapp needs an oracle so that the results of external sports event data are safely delivered to the blockchain. It is clear that if the oracle is malicious or provides wrong information, the execution of the smart contracts based on the external information is trustless.

### 2.3. Intel SGX

Intel SGX [21] is an extended x86 instruction set developed by Intel. Intel SGX allows a user program to be executed in an *enclave*, a memory area that other user programs and operating systems cannot access. When a user attempts to access the enclave memory area through a normal function call or command, the CPU cancels it and allows access only when the function is called from the enclave’s internal code. In other words, by restricting access to the enclave’s internal memory from outside the enclave, Intel SGX ensures the integrity and confidentiality of program execution within the enclave.

However, only the area allocated to the enclave is secure and therefore the execution such as a system call that needs to switch to kernel mode cannot be performed inside the enclave. For this reason, Intel SGX-based programs are divided into *trusted zone* and *untrusted zone*. While functions that require integrity are usually implemented in the trusted zone, functions that do not require system calls or perform flawlessly are implemented in the untrusted zone.

**Figure 2:**
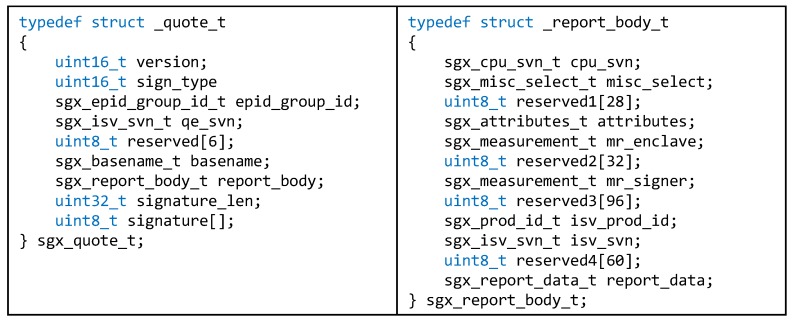
Data Structure for Remote Attestation in Intel SGX.

Another feature of Intel SGX is the support of remote attestation. Remote attestation proves to the remote entity that a running program is safely executed, and it is actually intended by providing a hash of the code and data within the program. For example, the structure of *quote* and *report*, used for the remote attestation in Intel SGX, is shown in Figure 2. An enclave that needs the remote attestation first passes the *quote* structure (i.e., *sgx_quote_t*) including the *report* structure (i.e., *sgx_report_body_t*) to the remote enclave by encrypting the contents with a private key accessible only by the original enclave. Then, the remote enclave decrypts the received contents with a group public key, verifies the validity of the *quote*, and sends the result back to the original enclave. When the remote enclave checks the validity, the 256-bit hash value *mr_enclave* in *sgx_report_body_t* is used. This hash value summarizes the code and initial data for the original enclave.

### 2.4. Motivation

To develop secure and robust blockchain-based IoT Dapps, the blockchain oracle should support data availability and data integrity. The communication channel between an oracle server and external sources also need to be secured.

For ensuring data availability, the blockchain oracle should be designed so that it does not suffer from a single point of failure problem. One of the most efficient ways to achieve this is to build multiple oracle servers and make them continuously running even when an oracle server fails. Moreover, the blockchain oracle needs to support data integrity. It must be verified that the external data is safely received and is not tampered before transferring to the blockchain. Both TLS and Intel SGX are possible solutions to prevent external data from being tampered. While TLS provides privacy and data integrity between an oracle and external data sources, Intel SGX is a hardware security solution that allows code and data within an enclave to be safely executed.

An oracle server with TLS and Intel SGX can create another problem in the blockchain-based IoT Dapps where an oracle server fetches time-variant IoT data from external sources. As the time-variant IoT data is collected periodically through multiple IoT sensors, the value of data changes so rapidly and the volume of data is sometimes huge.

To explain this further, we take an example from Coldchain [25], which is a temperature-controlled supply chain that typically includes equipment technologies such as packaging, temperature-controlled containers, transportation vehicle and warehouse facilities.

In a blockchain-based IoT Dapp for Coldchain, a blockchain oracle needs to collect the temperatures of containers using IoT sensors and send the information back to the blockchain. If the temperatures of containers are higher than a certain upper limit, a penalty is issued to the company managing the containers.

**Figure 3:**
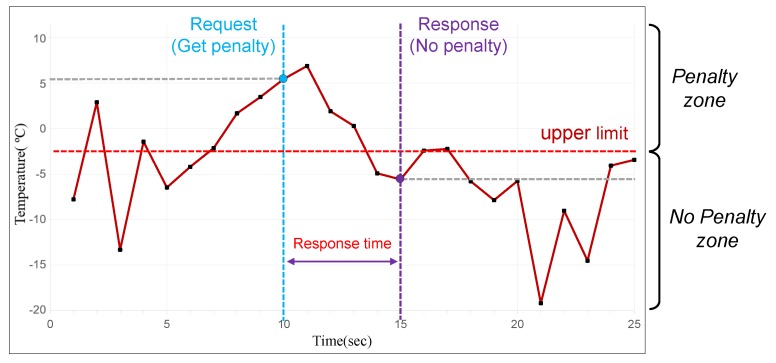
Time-Variant IoT Data in Coldchain.

Figure 3 shows the temperature data of a Coldchain container for 25 s. Assume that the upper limit is set to −2 °C. Thus, the area where the temperatures of the containers are higher than this limit is called the penalty zone, and in the opposite case, the no penalty zone. Assume also that the blockchain oracle receives a data request from the blockchain after 10 s and the temperature at the time when the request has arrived is in the penalty zone (around +5 °C). However, if a certain amount time (i.e., 5 s response time) is required to provide data due to server overload, the temperature data given to the blockchain is around −5 °C, which is in the no penalty zone. This results in a situation where the company that is supposed to get a penalty is not penalized as shown in Figure 3. Therefore, to provide correct external data, the response time must be minimized, which also indicates the performance of an oracle server. This problem also happens when a malicious oracle node causes a data abusing problem by deliberately delaying the response. A special mechanism should be devised to solve this problem, too.

DiOr-SGX has been designed so that it supports data availability by using multiple oracle servers and data integrity by using TLS and Intel SGX. DiOr-SGX also solves the response time problem and the data abusing problem by using a reputation system that excludes a malicious or overloaded oracle server as much as possible.

## 3. Related Work

There have been many research activities to solve the blockchain oracle problem. Existing approaches are largely classified into either *centralized* or *distributed*.

A centralized oracle is an oracle running on a single server such as Oraclize [9] and Town-crier [10]. Oraclize is a trusted third party that has been designed to provide external data to the blockchain. Oraclize is one of the first oracle protocols for Ethereum. When importing external data through Uniform Resource Locator (URL) or Application Programming Interface (API), Oraclize uses TLS-notary [26] to fetch data safely. However, the blockchain layer in the Oraclize cannot confirm the integrity of the received data since the oracle code runs in the untrusted zone. Town-Crier is also a centralized oracle that uses a trusted hardware called Intel SGX for data integrity. Although both approaches provide a certain level of data integrity, they fail to support data availability. Moreover, they are also prone to cause data abusing problem because their protocols are implemented over a single oracle node.

A distributed oracle is an oracle where multiple oracle servers form a peer-to-peer network to provide external data to the blockchain such as ASTRAEA [11], Shintaku [12], and Chainlink [13]. ASTRAEA is the first distributed oracle protocol to solve blockchain oracle problem. For each oracle data request, ASTRAEA provides external data through true–false votes on questions raised by the blockchain. ASTRAEA is based on the assumption that voters will only vote sincerely without a special reward or penalty. In addition, ASTRAEA cannot guarantee data integrity because no security facilities such as Intel SGX or TLS are used to import external data. Shintaku is another distributed oracle using the same true–false-based voting system as ASTRAEA. Shintaku encourages voters to participate and vote correctly by providing reward or penalty for oracle questions. However, as with ASTREA, data integrity is not guaranteed because it runs without any security facilities. Chainlink is a distributed oracle that configures the oracle layer as a blockchain. Unlike other approaches, the external data imported by each oracle server is agreed through the Byzantine Fault Tolerance (BFT) consensus algorithm [27]. A reward is given to the node that provides the agreed data in the form of a coin. Chainlink also does not support data integrity due to the absence of security properties.

As a distributed oracle runs with multiple oracle nodes, the data availability problem due to single point of failure can be resolved. However, the true–false-based voting system makes the oracle difficult to reach a consensus on the values for time-variant IoT data. In addition, existing distributed oracles are configured with a relatively small network size (i.e., number of participating oracle nodes), and small rewards or penalties. Potentially, this may result in many malicious oracle nodes and therefore can cause data integrity problem because malicious oracle nodes are vulnerable to a 51% attack. Finally, it is not possible for existing oracle protocols to guarantee a relatively consistent response time. The response time affects the overall performance of the oracle and the oracle protocol should consider how to minimize it.

Table 1 summarizes the comparison between existing oracle protocols and DiOr-SGX which is proposed in this paper.

## 4. Design and Implementation

This section presents the overall architecture of DiOr-SGX, and discusses its design and implementation issues in detail.

### 4.1. Overall Architecture

**Figure 4:**
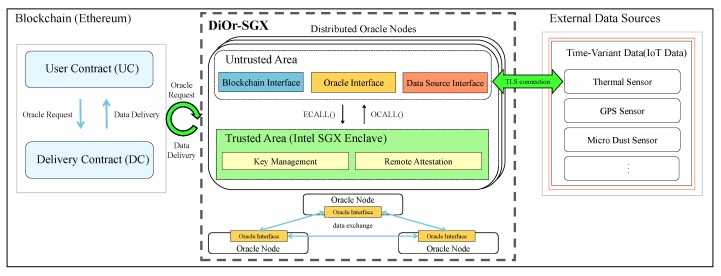
Overall Architecture of DiOr-SGX.

Figure 4 shows the overall architecture of DiOr-SGX. DiOr-SGX consists of multiple distributed oracle nodes, where each oracle node contains software components both in a trusted area and in an untrusted area. The trusted area includes software components that need to be executed safely such as key management and remote attestation. DiOr-SGX uses Intel SGX to implement those functions in a trusted area called enclave. For example, the data from external data sources is obtained through TLS communication and a consensus is reached among distributed oracle nodes through remote attestation inside an enclave. However, the untrusted area includes software functions that cannot be performed inside an enclave such as software interfaces to Ethereum blockchain, other oracle nodes and external data sources. The communication between the two areas is done by Intel SGX’s trusted library functions (ECALL and OCALL).

In order for Ethereum Dapp users to interact with DiOr-SGX, two smart contracts such as *user contract* (UC) and *delivery contract* (DC) are necessary. The UC is a smart contract developed by Ethereum Dapp users to request data from external data sources. The DC which is invoked from the UC interacts with DiOr-SGX to request or receive external data. The TLS connection between DiOr-SGX and external data sources is established to ensure the privacy and data integrity of the received data. The vulnerability of a smart contract is an important issue for blockchain systems. However, this paper assumes that all input smart contracts have no vulnerabilities during execution.

**Figure 5:**
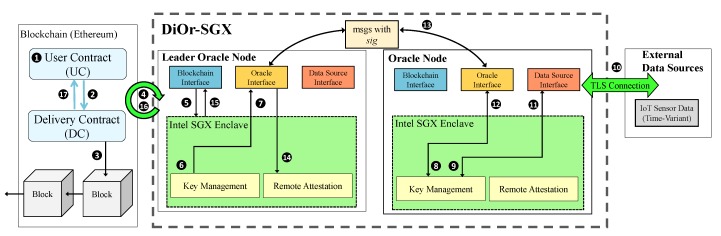
Delivery Process of Oracle Request and External Data.

Figure 5 depicts how the oracle requests from Ethereum Dapp users are delivered to external sources and how the external data is finally delivered back to the users.

Initially, ① any Ethereum Dapp user who wants to obtain external data creates an UC. ② When the UC needs to access external data, it invokes the DC. ③ Then, the DC with the request generate a block as an event log. ④ When the blockchain interface in the leader oracle node of DiOr-SGX detects the event log, ⑤ the request is delivered to the enclave. ⑥ After the message signature including the request is calculated inside the enclave, ⑦ then the message with the signature is broadcast to other oracle nodes using the oracle interface. ⑧ Other oracle nodes then verify the received message inside their enclaves and ⑨ ⑩ use TLS communication to get data from external data sources through data source interface.

On the other hand, ⑪ the data delivery process from external sources starts with each oracle node generating a signature of received IoT data inside its enclave and ⑫ ⑬ transferring the data with the signature to the leader oracle node. ⑭ When the leader oracle node completes data reception, it validates the signature of the received data through remote attestation and ⑮ ⑯ sends the average of the received data with signature to the DC as a transaction. ⑰ Finally, the DC verifies the received transaction and delivers external data to the UC as a callback function.

### 4.2. Delivery Contract

A delivery contract (DC) is an intermediary between the user contract (UC) requesting external data and DiOr-SGX. The DC is a smart contract in Ethereum that is responsible for forwarding oracle requests, electing a leader oracle node, checking the validity of a message, and managing the reputation of each oracle node.

**Figure 6:**
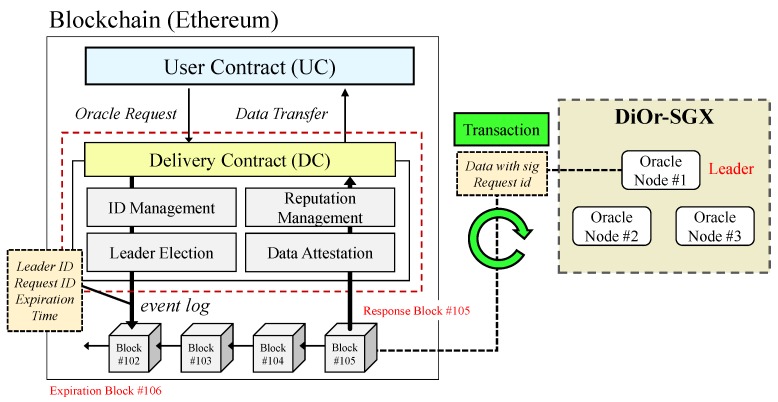
Delivery Contract Operation.

Figure 6 shows how the DC interacts with DiOr-SGX in detail. When an oracle request is initially issued from the UC developed by an Ethereum Dapp user, the DC is invoked. Then, the DC assigns a unique ID to the request and selects a leader oracle node based on the reputation of all oracle nodes. After this, an Ethereum block is generated as an event log to inform DiOr-SGX that Ethereum Dapp has an oracle request. The parameters recorded in the block include an ID of elected leader oracle node, an ID given to the oracle request, and expiration time. The expiration time is a time limit for the DC to receive external data from a leader oracle node, which is measured based on the block height. For example, if the elected leader oracle node does not respond until a certain number of blocks are generated (i.e., block height), it is considered to be offline or a malicious node that does not provide data intentionally. Since the reputation of a leader oracle node is largely depends on its response time, this leader oracle node is likely to be excluded from the possible candidates.

However, when DiOr-SGX obtains data from external data sources, it generates a transaction that includes external data with signature and request ID, which is delivered to the DC. After the DC checks the validity of data and request ID, it updates the reputation of the leader oracle node using response time and sends external data back to the UC through a call back function.

### 4.3. Securing Oracle Node Using Intel SGX

All DiOr-SGX features requiring safety are implemented within the Intel SGX enclave. These are functions such as key management and remote attestation.

The key management module manages a unique asymmetric key pair created based on a random seed. In this asymmetric key pair, the private key is used to encrypt data inside the enclave and the public key is used to decrypt the data encrypted with the private key. The public key is included in *report_data* field of *report* structure (i.e., *sgx_report_body_t*) as shown in Figure 2. In DiOr-SGX, all oracle nodes in the same oracle network exchange each other’s public keys and register their public keys in the DC. Therefore, the Ethereum Dapp users are aware that the transaction delivered by the oracle node is created inside the enclave through the registered public keys. In addition, DiOr-SGX proves to other nodes that the key pair is created inside the enclave, and there is no code leaking the private key to the outside. After the leader oracle node receives external data from other nodes, remote attestation is required to confirm that other oracle nodes have safely obtained the data. DiOr-SGX uses the remote attestation services provided by Intel SGX.

**Figure 7:**
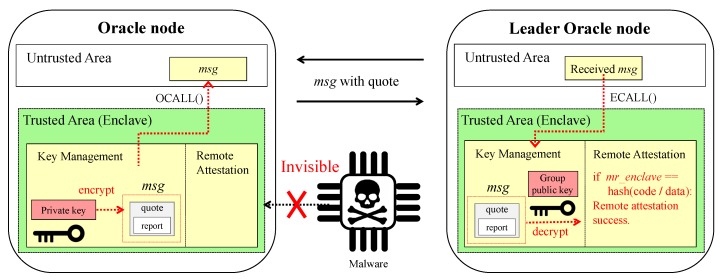
Remote Attestation in DiOr-SGX.

Figure 7 shows the process of remote attestation in DiOr-SGX. As shown in Figure 7, remote attestation in DiOr-SGX starts with the oracle node making the *quote* structure (i.e., *sgx_quote_t*) based on the code and data used for receiving external data. Then, each oracle node encrypts the *quote* structure with its private key inside its enclave and sends a message including the *quote* structure to the leader oracle node through oracle interface. After the leader oracle node receives the message, it decrypts the message using a group public key inside its enclave. Thereafter, the leader oracle node completes the remote attestation by determining whether the *mr_enclave* field included in the *report* structure matches with the hash value of the executed code and data.

### 4.4. Reputation Management and Leader Election

As explained in Section 4.2, the DC is responsible for electing a leader oracle node and managing its reputation, which is largely based on the response time of each oracle node. An oracle node with a shorter response time has better reputation and is likely to be chosen as a leader oracle node. Equations (1) through (5) explain how the reputation of a leader oracle node is calculated.

(1) $$RT_{leader}=\left(Block\;{\#}_{response}-Block\;{\#}_{request}\right)\times Blockinterval$$

(2) $$AvrgRT=\frac{1}{N_{oracle}}\times \sum_{i=1}^{N_{oracle}}RT_{i}$$

(3) $$R_{leader}=\frac{AvrgRT}{RT_{leader}}$$

(4) $$C_{leader}=min\left(\frac{N_{response}}{N_{limit}},1\right)$$

(5) $$new\;Rep_{leader}=old\;Rep_{leader}\times R_{leader}\times C_{leader}$$

First of all, the response time of a leader oracle node, $RT_{leader}$, is calculated by multiplying the difference between the block numbers when an oracle request and its response are included, by the block interval Blockinterval as shown in Equation (1). Then, the average response time, AvrgRT, which is the sum of response times from all oracle nodes divided by the number of oracle nodes, $N_{oracle}$, is calculated as shown in Equation (2). Using $RT_{leader}$ and AvrgRT, the reward value of a leader oracle node, $R_{leader}$, which indicates the goodness of a leader oracle node’s response time is obtained as shown in Equation (3). If $RT_{leader}$ is shorter than AvrgRT, bigger reward is given to the leader oracle node. $C_{leader}$ shown in Equation (4) is the level of confidence of the response time from a leader oracle node calculated by $N_{response}$ and $N_{limit}$. While $N_{response}$ represents the actual number of responses a leader oracle node receives from other oracle nodes, $N_{limit}$ is a pre-defined number of responses set by the administrator. As a result, the case where $N_{response}$ is larger than $N_{limit}$ is more favorable than the opposite case. Finally, the reputation of a leader oracle node, $newRep_{leader}$, is updated based on the leader’s previous reputation, $oldRep_{leader}$, multiplied by $R_{leader}$ and $C_{leader}$ of the leader oracle node as shown in Equation (5). The reputation value of each oracle node is used to elect a leader oracle node.

In order for an oracle node with larger reputation value to get a better chance of being elected as a leader oracle node, a special random function is designed in DiOr-SGX since it is impossible to generate random numbers in Ethereum’s smart contract. The random function creates a random number by a *modulo* operation with the sum of each block’s block hash (i.e., block.number-1) and reputation of each oracle node. Then, the number is subtracted by the reputation value of each oracle node until it becomes less than 0. If the number becomes less than 0 in the *i*-th oracle node, the node is elected as a leader oracle node.

**Figure 8:**
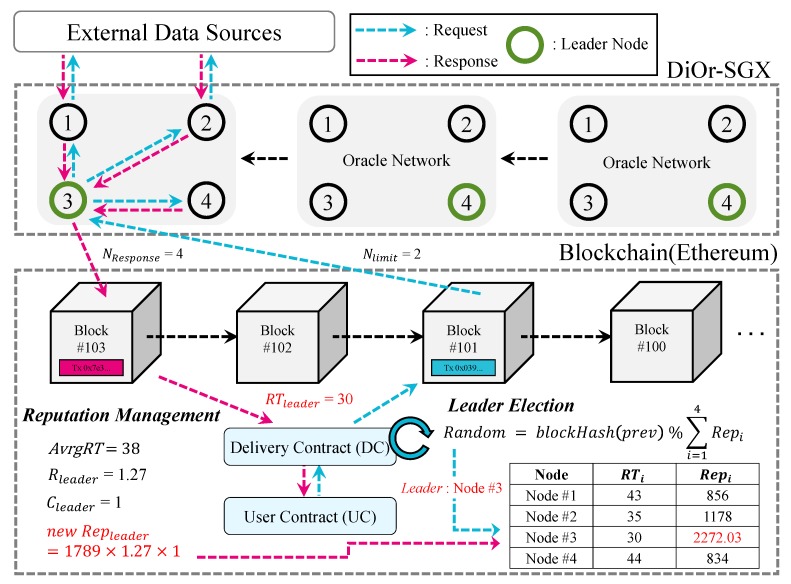
Example of Reputation Management and Leader Election.

Figure 8 is a detailed example that describes how the reputation of each oracle node is calculated and how a leader oracle node with higher reputation value is more likely to be selected as a leader. We assume that the DC issues an oracle request at the 101-th block and receives its response at the 103-th block from a leader oracle node. Current leader oracle node is *Node* #3 and the Blockinterval is set to 15 s because the block interval in Ethereum is between 10 to 20 s. We also assume that the values for $N_{oracle}$ and $N_{limit}$ are set to 4 and 2, respectively. Moreover, the reputation management table shown in the bottom right-hand corner summarizes the response time of each oracle node and the corresponding reputation value.

Then, based on the assumption, the response time of *Node* #3, $RT_{leader}$, is 30 s. Since *Node* #3 receives the responses from all oracle nodes, $N_{response}$ is 4 and the average response time, AvrgRT, is calculated as 38 s according to the reputation management table. As a result, the reward value $R_{leader}$ of *Node* #3 becomes 1.27 because the response time of *Node* #3 is shorter than the average. The confidence value $C_{leader}$ of *Node* #3 is 1 because the number of responses, $N_{response}$, received by *Node* #3 is larger than the pre-defined number of responses $N_{limit}$. If the previous reputation value of *Node* #3 is 1789, the new reputation value is increased to 2272.03 and updated in the reputation management table.

Now that the reputation value $Rep_{i}$ of each oracle node is 856, 1178, 2272.03, 834, the random number becomes a value between 0 and 5139. Considering that the *Node* #3 has larger reputation value than other nodes, the chance of *Node* #3 being elected as a leader oracle node increases. Algorithm 1 is a pseudocode that explains the reputation management and leader election process in DiOr-SGX.
**Algorithm 1:** Reputation Management and Leader Election Algorithm **Require:**  *blockHash*(*previous*): Hash of previous block  *block*#(*response*): Block number of response time  *block*#(*request*): Block number of request time  *Rep_i_*: Reputation of each oracle node **procedure**
Reputation_Management():1: sum ← 0, i ← 12: *RT_leader_* = (*block*#(*response*) − *block*#(*request*)) × *blockInterval*3: **while**
*i* ≤ *N_oracle_*
**do**4:  *sum* += *RT_i_*5:  *i* ≤ *i* + 1  **end**6: *AvrgRT* = *sum*/*Noracle*7: *R_leader_* = *AvrgRT*/*RT_leader_*8: *C_leader_* = *min*(*N_response_*/*N_limit_*, 1)9: *Rep_leader_* = *Rep_leader_* × *R_leader_* × *C_leader_*10: **procedure**
Leader_Election():11: *sum* ← 0, i ← 112: **while**
*i* ≤ *N_oracle_*
**do**13:  *sum* += *Rep_i_*14:  *i* ← *i* + 1  **end**15: *mod* = *blockHash*(*previous*)%*sum*16: **while**
*mod* ≥ 0 **do**17:  *mod* = *mod* − *Rep_i_*18:  **if**
*mod* < 0 **then**19:   *i*-th oracle node is elected as a leader node   **end**20:  *i* ← *i* + 1  **end**


## 5. Performance Evaluation

### 5.1. Experiment Setup

DiOr-SGX is implemented over a cloud-based trusted execution environment (TEE) provided by Microsoft as part of Azure confidential computing efforts [28]. Open enclave SDK [29] is used to develop Intel SGX functions in DiOr-SGX. Each oracle server runs on a virtual machine (VM) with Intel SGX support. Each VM is configured to run with 4 vCPUs and 8 GB RAM. The servers run Ubuntu 18.04.3 LTS operating systems. The workload used for the experiment is ultra-fine dust data for 24 h which is real IoT data measured in Seoul [30].

For the evaluation, we first compare the performance of DiOr-SGX with Town-crier which is a centralized oracle server with Intel SGX support. For this, the response time for 1000 oracle requests is measured by varying the number of oracle severs from 3 to 9. The pre-defined number of oracle responses, $N_{limit}$, is set to 50% of the number of oracle servers. The purpose of this experiment is to compare the response time of DiOr-SGX with a centralized oracle with Intel SGX, and thus check the overhead of DiOr-SGX incurred by having multiple oracle servers. In addition, we also report the performance results of DiOr-SGX’s reputation system by intentionally creating malicious oracle nodes.

### 5.2. Evaluation of Response Time

**Figure 9:**
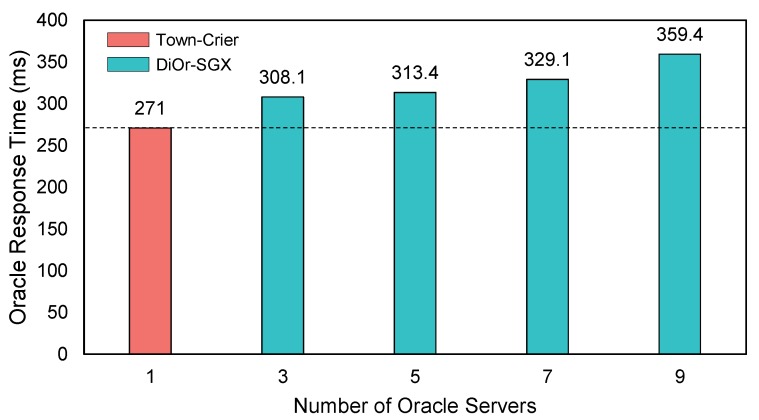
Comparison of Response Time (Town-crier vs DiOr-SGX).

Figure 9 shows the response times of DiOr-SGX and its comparison with Town-crier when the number of oracle servers is increased from 3 to 9. The measured response time excludes the time taken in Ethereum blockchain since the block generation time (i.e., 15 s) is too huge to exactly reflect the overall response time. Therefore, we measure the time from when the ECALL for an oracle request is issued in DiOr-SGX to when an Ethereum transaction is generated to return external data to the DC.

As shown in Figure 9, the performance of DiOr-SGX seems to be worse than that of Town-crier, which is apparent considering that Town-crier is a centralized oracle supporting Intel SGX and thus does not have collaborations among oracle servers. For example, the response time of Town-crier is 271 ms, whereas the response times of DiOr-SGX are 308.1 ms, 313.4 ms, 329.1 ms, 359.4 ms, when the number of servers is 3, 5, 7, 9, respectively.

To analyze this further, we have measured the time taken by each of the sub-tasks in a request-response process such as the times for message generation, message validation, transaction generation and TLS connection. The response time breakdown is shown in Table 2. As shown in Table 2, about 73% to 92% of the response time is consumed during TLS connection, and the time spent for other activities is minimal. The times taken for message generation and validation get larger because the number of messages generated and validated by a leader oracle node increases as the number of oracle servers increases. However, the time taken for transaction generation remains almost the same regardless of the number of oracle servers since only a leader oracle node is responsible for generating a transaction.

It is worthy to note that the time to get external data using TLS connection is one of the most dominant factors that affect the overall response time for all server configurations. If we exclude the TLS connection time from the response time, the performance difference between Town-crier and DiOr-SGX is negligible. The performance of DiOr-SGX does not significantly degrade as we increase the number of oracle servers. This indicates that DiOr-SGX can guarantee data availability and data integrity better than Town-crier with few performance disadvantages even when the number of nodes increases. On the other hand, if a transaction to the DC occurs within a normal block interval in Ethereum (i.e., 15 s), external data can be included in the next block immediately. Therefore, for a single data provision, both DiOr-SGX and Town-crier can provide external data to the next block, and thus there is no difference in performance.

### 5.3. Evaluation of Reputation System

One of the most important design philosophies in DiOr-SGX is to minimize response time as much as possible and provide an efficient mechanism to reduce the possibility that malicious oracle nodes are selected as a leader oracle node. To achieve this, DiOr-SGX’s reputation system is designed such that a leaders oracle node with a faster response time is favored and it is more likely to be chosen as a leader oracle node.

**Figure 10:**
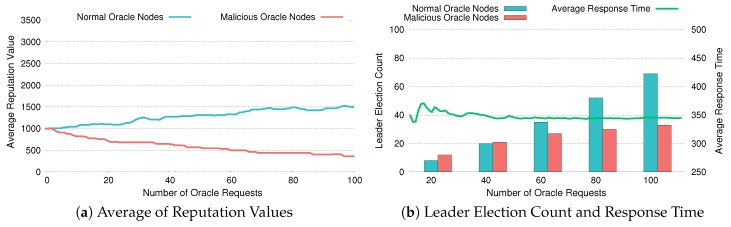
Evaluation of Reputation with 120% Response Time.

**Figure 11:**
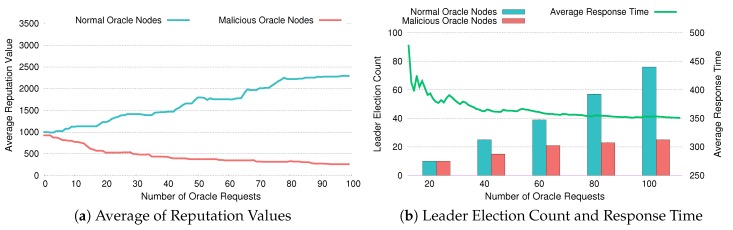
Evaluation of Reputation with 150% Response Time.

**Figure 12:**
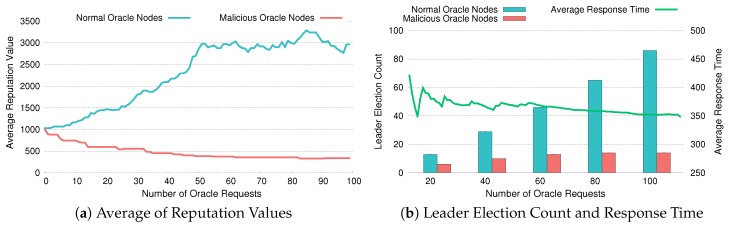
Evaluation of Reputation with 200% Response Time.

Figure 10, Figure 11 and Figure 12 depict whether DiOr-SGX’s reputation system is working properly and how it reacts when some of the oracle servers are malicious. Especially in Figure 10a, Figure 11a and Figure 12a, each figure shows the average reputation values of normal oracle nodes and malicious oracle nodes as the number of oracle requests is increased. In Figure 10b, Figure 11b and Figure 12b, each figure shows the accumulated leader election counts and average response times of both types of oracle nodes.

For the experiment, we assume that $N_{oracle}$, the total number of oracle servers, is 9 and almost half of them (i.e., 4 nodes) are malicious. Also, we assume that the total number of oracle requests is 100 and the default reputation value is set to 1000, which can be increased infinitely and cannot be less than zero. To figure out the mechanism used in DiOr-SGX’s reputation system and how it reduces average response time, we have conducted 3 experiments. In these experiments, the response times of malicious nodes are increased by 120%, 150%, and 200%, respectively to reflect a situation where malicious nodes are abusing data. If response time gets longer, malicious nodes are abusing data more heavily.

Figure 10 shows the results of an experiment in which the response times of malicious oracle nodes are 120% above the average. As shown in Figure 10a, after processing 100 oracle requests, the average reputation of normal oracle nodes rises up to about 150% of the default, while the average reputation of malicious oracle nodes drops down to about 36% of the default. This indicates that DiOr-SGX’s reputation system favors the nodes with short response times (i.e., normal oracle nodes) more than those with long response times (i.e., malicious oracle nodes). Furthermore, as shown in Figure 10b, the accumulated election count of normal nodes as a leader oracle becomes larger compare to that of malicious oracle nodes. For example, by the time when 100 oracle requests are processed, normal oracle nodes are elected as a leader oracle 68 times while malicious nodes are elected as a leader oracle 32 times. Although it is a small amount, the average response time of all oracle nodes also decreases.

Similarly, Figure 11 and Figure 12 show the results of experiments where the response times of malicious oracle nodes are 150% and 200% above the average, respectively. As expected, the average reputation keeps increasing as more oracle requests are processed. The difference between the average reputation of normal oracle nodes and that of malicious oracle nodes becomes larger as the response times of malicious oracle nodes get increased from 150% to 200%. For example, as shown in Figure 11a, after 100 oracle requests, the average reputation of normal oracle nodes is about 229% of the default, while the reputation of malicious oracle nodes is 26% of the default. However, in a case where the response time is increased by 200%, the corresponding values are 296% and 33% of the default as shown in Figure 12a. This means that as malicious nodes abuse data more heavily, the reputation of normal oracle nodes increases more rapidly, which means that normal oracle nodes are more likely to be selected as a leader node. In the experiments for getting accumulated leader election count and average response time as shown in Figure 11b and Figure 12b, we can also notice the same trend. When the average reputation of normal oracle nodes is overwhelmingly high, fewer malicious nodes are selected. Therefore, the average response time is also decreased.

## 6. Conclusions

In this paper, we have proposed a distributed oracle using Intel SGX called DiOr-SGX for blockchain-based IoT applications. By building multiple oracle servers, DiOr-SGX minimizes the problems caused by data abusing and single point failure. It also guarantees data availability. In addition, DiOr-SGX guarantees data integrity using Intel SGX enclave and TLS connection. DiOr-SGX’s reputation system reduces the response time of time-variant external IoT data by making the oracle nodes with faster response time to be selected as a leader oracle node as much as possible.

Through experiments, the performance of DiOr-SGX has been measured and compared with Town-crier, a centralized oracle server with Intel SGX. Although the response time of DiOr-SGX with 3 oracle servers is worse than that of Town-crier by about 14%, this performance gap is not significant considering that Town-crier is a single node solution. Furthermore, the 15-s block interval in Ethereum hides most of the performance difference in milliseconds from the overall response time. The reputation system of DiOr-SGX has also been evaluated. The results show that DiOr-SGX minimizes the response time by excluding malicious nodes with long response time as much as possible.

DiOr-SGX is currently a blockchain oracle especially targeting at Ethereum. Since DiOr-SGX is a middle-ware solution that provides external data safely to the blockchain, it can be easily applied to other blockchain platforms without much modification. For example, the blockchain platforms with higher Transaction Per Second (TPS) than Ethereum such as Hyperledger Fabric [31] and IOTA [32] are good candidates for DiOr-SGX. Moreover, DiOr-SGX can be used to implement various real world IoT applications. Although current evaluation of DiOr-SGX uses a real world ultra-fine dust IoT data in Seoul, the evaluation with other practical IoT applications makes the impact of the proposed idea stronger.

## Figures and Tables

**Table 1:** Summary of Comparison for Oracle Protocols.

Oracle	Data	Data	Support Time	Guarantee
Protocols	Availability	Integrity	Variant Data	Response Time
Oraclize [9]	×	△	◯	×
Town-crier [10]	×	◯	△	×
ASTRAEA [11]	◯	×	×	×
Shintaku [12]	◯	×	×	×
Chainlink [13]	◯	×	×	×
DiOr-SGX (proposed)	◯	◯	◯	◯

**Table 2:** Response Time Breakdown (N = Number of Oracle Servers, Unit: Time (ms)/Ratio (%)).

Activity	Town-Crier		N = 3		N = 5		N = 7		N = 9	
	Time	Ratio	Time	Ratio	Time	Ratio	Time	Ratio	Time	Ratio
Msg Generation	0	0	20	6.5	32	10.2	49	14.8	65	18.0
Validation	0	0	4.1	1.3	6.4	2.0	9.1	2.7	10.4	2.8
Tx Generation	20	7.4	20	6.5	19	6.0	19	5.7	19	5.2
Subtotal	20 ms		44.1 ms		57.4 ms		77.1 ms		94.4 ms	
TLS connection	251	92.6	264	85.7	256	81.7	252	76.5	265	73.8
Total	271 ms		308.1 ms		313.4 ms		329.1 ms		359.4 ms	
